# Supplementary material for: Analysis of PPARγ Signaling Activity in Psoriasis
Source: Int J Mol Sci. 2021 Aug 10;22(16):8603. doi: 10.3390/ijms22168603 (PMC8395241; doi:10.3390/ijms22168603)
Supplement: Supplementary file 1 [file ijms-22-08603-s001.zip › Supplemental materials_Analysis of PPARg signaling activity in psoriasis/Pathway models/Models images and html files/Model of PPARG related pathways in psoriasis (short version)/5136.html]

GNAI2


# Protein GNAI2

|  |  |
| --- | --- |
| URN | urn:agi-llid:2771 |
| Total Entities | 0 |
| Connectivity | 958 |
| Name | GNAI2 |
| Description | guanine nucleotide binding protein (G protein), alpha inhibiting activity polypeptide 2 |
| Notes | The protein encoded by this gene is an alpha subunit of guanine nucleotide binding proteins (G proteins). The encoded protein contains the guanine nucleotide binding site and is involved in the hormonal regulation of adenylate cyclase. Several transcript variants encoding different isoforms have been found for this gene. [provided by RefSeq, Sep 2013] |
| Primary Cell Localization | Cytoplasm |

---

|  |  |
| --- | --- |
| Pathway | Serotonin Receptors Signaling |
|  | CXC Chemokine Receptor Signaling |
|  | Proplatelet Maturation |
|  | Eicosanoids in Myeloid Cell Activation |
|  | Eosinophil Activation |
|  | Mast-Cells De Novo Synthesized Mediators via IgE Independent Signaling |
|  | Neutrophil Recruitment and Priming |
|  | Neutrophil Degranulation via FPR1 Signaling |
|  | Neutrophil Chemotaxis |
|  | Potassium Flux Regulation |
|  | Nociception Expression Targets Signaling |
|  | Vascular Motility |
|  | Ca2+ Flux Regulation |
|  | NPY1R -> CRH/POMC Production |
|  | CRH -> Synthesis of Corticosteroids |
|  | ADORA3 -> Mast-Cell Degranulation |
|  | BDKRB1/2 -> Vasodilation |
|  | BDKRB1/2 -> Ion Channels |
|  | BDKRB1/2 -> Prostaglandin Generation |
|  | BDKRB1/2 -> Interleukins Production |
|  | CNR1/2 -> Membrane Transport |
|  | CNR1/2 -> Vascular Motility |
|  | CNR1/2 -> IL1B/2/4/6/10 Production |
|  | GALR1/2/3 -> POMC/NPY Production |
|  | GALR1/2/3 -> Neurotransmitter Metabolism |
|  | CHRM1 -> IL2 Production |
|  | CHRM1/2/3/5 -> Ion Channels |
|  | CHRM1/2/3 -> Vascular Motility |
|  | OPRD/OPRM -> Ion Channels |
|  | HTR1 -> IL6 Production |
|  | HTR1 -> Membrane Transport |
|  | HTR1 -> Vascular Motility |
|  | GRM2-4/6-8 (Presynaptic) -> Glutamate Release Attenuation |
|  | P2RY2/12/13/14 -> N-Type Calcium Channel |
|  | Proteins Involved in Arterial Hypertension |
|  | Contraction Due Vasospasm |
|  | Melanoma |
|  | Oxidative Stress in Vitiligo |
|  | Vitiligo |
|  | Eosinophil Activation and Degranulation in Asthma |
|  | Mast-Cell Related Chemokines Signaling in Asthma |
|  | Vascular Smooth Muscle Cell Vasodilation in Brain |
|  | NO Effect on Vasodilation and Vasoconstriction |
|  | MIF Role in Cholesteatoma |
|  | Cholesteatoma |
|  | Acetylcholine Expression Targets |
|  | CXCL6 Expression Targets |
|  | CXCL1 Expression Targets |
|  | CXCL2 Expression Targets |
|  | CXCL3 Expression Targets |
|  | CXCL5 Expression Targets |
|  | FPR1 -> Cytoskeleton Signaling |
|  | EGFR Transactivation in Cancer and Non-Cancer Cells |
|  | Dronabinol/Anandamide Expression Targets |
|  | NPY Expression Targets |
|  | Ras-GRF Regulation Signaling |
|  | Model of PPARG signaling in psoriasis |
|  | Model of PPARG related pathways in psoriasis (short version) |
|  | 2\_1\_Eosinophilia and neutrophilia in asthma: eosinophil migration and activation |
|  | Model of PPARG signaling in psoriais (tested) |

---

|  |  |
| --- | --- |
| MedScan ID | 2771 |

---

|  |  |
| --- | --- |
| LocusLink ID | 2771 |
|  | 14678 |
|  | 81664 |

---

|  |  |
| --- | --- |
| Alias | H\_LUCA15.1 |
|  | Guanine nucleotide-binding protein G(i), alpha-2 subunit |
|  | guanine nucleotide binding protein (G protein), alpha inhibiting 2 |
|  | GBI2\_HUMAN |
|  | WUGSC:H\_LUCA16.1 |
|  | guanine nucleotide binding protein (G protein), alpha inhibiting activity polypeptide 2 |
|  | WUGSC:H\_LUCA15.1 |
|  | GTP-binding regulatory Gi alpha-2 chain |
|  | LOC102642216 |
|  | GTP-binding protein (G-alpha-i2) |
|  | OTTHUMP00000211067 |
|  | guanine nucleotide-binding protein G(i) subunit alpha-2 |
|  | guanine nucleotide binding protein, alpha inhibiting II |
|  | OTTHUMP00000211070 |
|  | G alpha inhibitory type 2 (a) |
|  | G(i), alpha II |
|  | OTTHUMP00000211066 |
|  | G protein alpha inhibiting activity polypeptide 2 |
|  | H\_LUCA16.1 |
|  | C76432 |
|  | g alpha inhibitory type 2 |
|  | G(i2) |
|  | G alpha i2 |
|  | Gnai2 |
|  | G protein alpha inhibiting activity polypeptide II |
|  | GBI2 |
|  | GTP-binding regulatory protein Gi alpha-2 chain (adenylate |
|  | Galphai2 |
|  | Gia |
|  | guanine nucleotide binding protein, alpha inhibiting 2 |
|  | G(i), alpha-2 |
|  | Adenylate cyclase-inhibiting G alpha protein 2 |
|  | G protein subunit alpha i2 |
|  | G alpha inhibitory type 2 (b) |
|  | Adenylate cyclase-inhibiting G alpha protein II |
|  | GNAI2B |
|  | Guanine nucleotide-binding protein (G protein), alpha-inhibiting |
|  | GIP |
|  | GTP-binding regulatory protein Gi alpha-2 chain |
|  | adenylate cyclase-inhibiting G alpha protein |
|  | Gnai-2 |

---

|  |  |
| --- | --- |
| GO ID | 0001664 |
|  | 0031683 |
|  | 0005525 |
|  | 0003924 |
|  | 0046872 |
|  | 0007213 |
|  | 0007186 |
|  | 0001973 |
|  | 0007189 |
|  | 0007193 |
|  | 0007188 |
|  | 0007049 |
|  | 0051301 |
|  | 0008283 |
|  | 0007214 |
|  | 0007194 |
|  | 0140199 |
|  | 2001234 |
|  | 0045955 |
|  | 1903614 |
|  | 0050805 |
|  | 0070374 |
|  | 0033864 |
|  | 0030335 |
|  | 0008284 |
|  | 0046628 |
|  | 2000179 |
|  | 0035815 |
|  | 0032930 |
|  | 0035810 |
|  | 1904707 |
|  | 0006457 |
|  | 0051924 |
|  | 0007584 |
|  | 0007165 |
|  | 0044297 |
|  | 0005813 |
|  | 0005737 |
|  | 0005829 |
|  | 0030425 |
|  | 0070062 |
|  | 1903561 |
|  | 0005834 |
|  | 0016020 |
|  | 0045121 |
|  | 0030496 |
|  | 0005654 |
|  | 0005886 |
|  | 0019001 |
|  | 0000166 |
|  | 0008016 |
|  | 0005856 |
|  | 0005634 |
|  | 0007216 |
|  | 0070382 |
|  | 0004871 |
|  | 0000186 |
|  | 0035556 |
|  | 0008152 |
|  | 0007596 |
|  | 0030168 |
|  | 0007268 |
|  | 0031982 |
|  | 0031234 |
|  | 0006184 |
|  | 0007243 |
|  | 0051927 |
|  | 0005624 |

---

|  |  |
| --- | --- |
| KEGG ID | hsa:2771 |
|  | mmu:14678 |
|  | rno:81664 |

---

|  |  |
| --- | --- |
| Organism | Homo sapiens {Organism urn:agi-taxid:9606} |
|  | Mus musculus {Organism urn:agi-taxid:10090} |
|  | Rattus norvegicus {Organism urn:agi-taxid:10116} |
|  | Homo sapiens |
|  | Mus musculus |
|  | Rattus norvegicus |

---

|  |  |
| --- | --- |
| Mouse chromosome position | 9 58.43 cM |
|  | 9 59.0 cM |

---

|  |  |
| --- | --- |
| OMIM ID | 139360 |
|  | 192605 |

---

|  |  |
| --- | --- |
| Rat chromosome position | 8q32 |

---

|  |  |
| --- | --- |
| Hugo ID | 4385 |
|  | HGNC:4385 |

---

|  |  |
| --- | --- |
| Human chromosome position | 3p21.31 |
|  | 3p21 |

---

|  |  |
| --- | --- |
| Swiss-Prot Accession | B3KP24 |
|  | B3KX51 |
|  | P04899 |
|  | P04899.3 |
|  | P08752 |
|  | P08752.5 |
|  | P04897 |
|  | Q45QN0 |
|  | P04897.3 |
|  | B3KTZ0 |
|  | B4DYA0 |
|  | B4E2X5 |
|  | Q6B6N3 |
|  | Q8IZ71 |
|  | Q3TXK7 |
|  | Q6P1C0 |
|  | Q6LCB5 |
|  | Q93020 |
|  | Q96C71 |
|  | Q8JZT4 |
|  | Q922Y6 |
|  | Q5EEY3 |
|  | Q5EEY4 |

---

|  |  |
| --- | --- |
| PIR ID | S02319 |
|  | B25889 |
|  | D27423 |

---

|  |  |
| --- | --- |
| GenBank ID | NC\_000003 |
|  | NM\_001282619 |
|  | NP\_001269548 |
|  | NM\_001282620 |
|  | NP\_001269549 |
|  | NM\_001282617 |
|  | NP\_001269546 |
|  | NM\_002070 |
|  | NP\_002061 |
|  | NM\_001282618 |
|  | NP\_001269547 |
|  | NM\_001166425 |
|  | NP\_001159897 |
|  | NG\_016002 |
|  | AC002077 |
|  | AH001470 |
|  | AAA35894 |
|  | CH471055 |
|  | EAW65051 |
|  | EAW65052 |
|  | EAW65053 |
|  | EAW65054 |
|  | EAW65055 |
|  | EAW65056 |
|  | KT584007 |
|  | U73166 |
|  | AAD12230 |
|  | U73169 |
|  | AAD12229 |
|  | X07854 |
|  | CAA30703 |
|  | AF493906 |
|  | AAM12620 |
|  | AK055574 |
|  | BAG51536 |
|  | AK096299 |
|  | BAG53252 |
|  | AK096595 |
|  | BAG53334 |
|  | AK126708 |
|  | BAG54363 |
|  | AK131062 |
|  | AK302330 |
|  | BAG63662 |
|  | AK304473 |
|  | BAG65287 |
|  | AY677118 |
|  | AAT78421 |
|  | BC012138 |
|  | AAH12138 |
|  | BC014627 |
|  | AAH14627 |
|  | BC016995 |
|  | AAH16995 |
|  | CR456783 |
|  | CAG33064 |
|  | DA528337 |
|  | DB044421 |
|  | HY037331 |
|  | J03004 |
|  | AAA52556 |
|  | X04828 |
|  | CAA28512 |
|  | P04899 |
|  | NC\_000075 |
|  | XM\_006511637 |
|  | XP\_006511700 |
|  | NM\_008138 |
|  | NP\_032164 |
|  | AC162905 |
|  | AL672219 |
|  | AK157998 |
|  | BAE34308 |
|  | AK159222 |
|  | BAE34909 |
|  | AK160186 |
|  | AK167388 |
|  | BAE39478 |
|  | AK177418 |
|  | AK178281 |
|  | AK178376 |
|  | AK178445 |
|  | AK179275 |
|  | AK179602 |
|  | AK179809 |
|  | AK183811 |
|  | AK183887 |
|  | AK184440 |
|  | AK185434 |
|  | AK185758 |
|  | AK188055 |
|  | AK191152 |
|  | AK192434 |
|  | AK192815 |
|  | AK197984 |
|  | AK202003 |
|  | AK206522 |
|  | AK206873 |
|  | AK207227 |
|  | AK210682 |
|  | AK212898 |
|  | AK215781 |
|  | AK216428 |
|  | AK219691 |
|  | BC006695 |
|  | AAH06695 |
|  | BC037130 |
|  | AAH37130 |
|  | BC065159 |
|  | AAH65159 |
|  | CF951009 |
|  | CJ134826 |
|  | M13963 |
|  | AAA37692 |
|  | S71213 |
|  | AAB30632 |
|  | S79607 |
|  | P08752 |
|  | NC\_005107 |
|  | XM\_006243858 |
|  | XP\_006243920 |
|  | NM\_031035 |
|  | NP\_112297 |
|  | AC\_000076 |
|  | AAHX01056979 |
|  | AC106346 |
|  | CH473954 |
|  | EDL77222 |
|  | AY899210 |
|  | AAX07753 |
|  | AY899211 |
|  | AAX07754 |
|  | BC078806 |
|  | AAH78806 |
|  | CO394955 |
|  | CR465951 |
|  | DQ120471 |
|  | AAZ23810 |
|  | DQ120472 |
|  | AAZ23811 |
|  | M12672 |
|  | AAA41260 |
|  | M17528 |
|  | AAA40824 |
|  | P04897 |
|  | NC\_018914 |
|  | AMYH02006766 |
|  | M20586 |
|  | M20587 |
|  | M20588 |
|  | M20589 |
|  | M20590 |
|  | M20591 |
|  | M20592 |
|  | M20593 |
|  | DQ892783 |
|  | ABM83709 |
|  | DQ895908 |
|  | ABM86834 |
|  | AC\_000031 |
|  | AAHY01081493 |
|  | AAHY01081494 |
|  | AAHY01081495 |
|  | AAHY01081496 |
|  | AAHY01081945 |
|  | AK185480 |
|  | AC\_000135 |
|  | ABBA01024553 |
|  | XM\_005265066 |
|  | XP\_005265123 |
|  | XM\_005265068 |
|  | XP\_005265125 |
|  | XM\_005265067 |
|  | XP\_005265124 |
|  | NW\_003871059 |
|  | XM\_006243859 |
|  | XP\_006243921 |
|  | AABR06057030 |
|  | NT\_022517 |
|  | NW\_001838877 |
|  | NW\_921651 |
|  | AC\_000046 |
|  | CR593068 |
|  | CR599468 |
|  | CR603761 |
|  | CR604433 |
|  | CR606737 |
|  | CR609921 |
|  | CR610539 |
|  | CR618994 |
|  | Q6B6N3 |
|  | Q6LCB5 |
|  | Q93020 |
|  | Q96C71 |
|  | NT\_039477 |
|  | NW\_001030918 |
|  | Q3TXK7 |
|  | Q8JZT4 |
|  | Q922Y6 |
|  | NW\_047801 |
|  | NW\_001084876 |
|  | Q45QN0 |
|  | Q5EEY3 |
|  | Q5EEY4 |
|  | NW\_001030920 |

---

|  |  |
| --- | --- |
| Swiss-Prot ID | GNAI2\_HUMAN |
|  | GNAI2\_MOUSE |
|  | GNAI2\_RAT |
|  | Q3TXK7\_MOUSE |
|  | B3KX51\_HUMAN |
|  | B4DYA0\_HUMAN |
|  | Q45QN0\_RAT |
|  | B3KP24\_HUMAN |

---

|  |  |
| --- | --- |
| Cell Localization | Cytoplasm |
|  | Cytoskeleton |
|  | Microtubule organizing center |
|  | Centrosome |
|  | Cell membrane |
|  | Membrane |
|  | Cytoplasm, cytoskeleton, microtubule organizing center, centrosome |

---

|  |  |
| --- | --- |
| Ensembl ID | ENSG00000114353 |
|  | ENSP00000406369.1 |
|  | ENST00000451956.1 |
|  | ENSP00000266027.6 |
|  | ENST00000266027.9 |
|  | ENSP00000406871.1 |
|  | ENST00000422163.5 |
|  | ENSP00000312999.6 |
|  | ENST00000313601.11 |
|  | ENSMUSG00000032562 |
|  | ENSMUSP00000057543.6 |
|  | ENSMUST00000055704.11 |
|  | ENSMUSP00000142326.1 |
|  | ENSMUST00000192615.5 |
|  | ENSRNOG00000016592 |
|  | ENSRNOP00000022550.4 |
|  | ENSRNOT00000022550.6 |
|  | ENSP00000406369 |
|  | ENST00000451956 |
|  | ENSP00000266027 |
|  | ENST00000266027 |
|  | ENSP00000406871 |
|  | ENST00000422163 |
|  | ENSP00000312999 |
|  | ENST00000313601 |
|  | ENSMUSP00000057543 |
|  | ENSMUST00000055704 |
|  | ENSMUSP00000142326 |
|  | ENSMUST00000192615 |
|  | ENSRNOP00000022550 |
|  | ENSRNOT00000022550 |

---

|  |  |
| --- | --- |
| MGI ID | MGI:95772 |
|  | 95772 |

---

|  |  |
| --- | --- |
| RGD ID | 620243 |

---

|  |  |
| --- | --- |
| Unigene ID | Hs.77269 |
|  | Mm.196464 |
|  | Rn.3036 |

---

|  |  |
| --- | --- |
| Homologene ID | 55539 |

---

|  |  |
| --- | --- |
| IPI ID | IPI00217906 |
|  | IPI00748145 |
|  | IPI00926935 |
|  | IPI01015661 |
|  | IPI00231925 |
|  | IPI00228617 |
|  | IPI00652902 |
|  | IPI00924427 |
|  | IPI00794986 |
|  | IPI00465121 |
|  | IPI00796250 |
|  | IPI00798195 |
|  | IPI00797567 |
|  | IPI00925691 |

---
